# Efficacy of Montelukast combined with Sublingual Immunotherapy in the treatment of children with Obstructive Sleep Apnea Hypopnea Syndrome Complicated with allergic rhinitis

**DOI:** 10.12669/pjms.39.5.6985

**Published:** 2023

**Authors:** Xiuhua Mao, Wenjuan Zhao

**Affiliations:** 1 Xiuhua Mao School of Health, Dongguan Polytechnic, Dongguan 523186, Guangdong, China; 2 Wenjuan Zhao Department of Pharmacy, Hunan Children’s Hospital, Changsha 410007, Hunan, China

**Keywords:** Montelukast, Sublingual immunotherapy, Obstructive sleep apnea hypopnea Syndrome, Allergic rhinitis

## Abstract

**Objective:**

To observe the efficacy of Montelukast combined with sublingual immunotherapy (SLIT) in the treatment of children with obstructive sleep apnea hypopnea syndrome (OSAHS) complicated with allergic rhinitis (AR).

**Methods:**

This is a prospective study. A total of 102 children with OSAHS complicated with allergic rhinitis admitted to Hunan Children’s Hospital from April 2020 to April 2021 were selected and randomly divided into two groups: sublingual group and two-drug combination group. Children in the sublingual group were treated with standardized dust mite drops Nos. 1-4 for SLIT, while those in the two-drug combination group were treated with Montelukast on top of the sublingual group. Statistical analysis and comparison were made between the two groups of children in terms of Sleep apnea hypopnea index (AHI), Hypoxic saturation (Lsao_2_), interleukin-4, Il-4), interleukin-17 (IL-17), OSA-18 Snoring Symptom Scale for Children (OSA-18), allergic rhinitis symptom scale (TNSS), efficacy, occurrence of adverse reactions, etc.

**Results:**

After treatment, the AHI index of the two-drug combination group was significantly decreased, and the Lsao_2_ index was significantly increased compared with the sublingual group (P<0.05). Compared with the sublingual group, the levels of IL-4 and IL-17 in the two-drug combination group were significantly decreased (P<0.05), the OSA-18 score and TNSS score were significantly lower (P<0.05). Moreover, compared with the sublingual group, the efficacy of the two-drug combination group was significantly increased (P<0.05), the incidence of adverse reactions was significantly lower (P<0.05).

**Conclusion:**

Montelukast combined with sublingual immunotherapy offers many advantages, such as effectively controlling nasal allergy symptoms in children with OSAHS complicated with allergic rhinitis and improving OSAHS symptoms.

## INTRODUCTION

Obstructive sleep apnea hypopnea syndrome (OSAHS) refers to the obstructive breathing caused by complete or partial upper airway obstruction during night sleep, resulting in snoring, dyspnea and mouth buccal breathing. It is often accompanied by symptoms such as sleep apnea. OSAHS severely affects the normal sleep structure of patients, manifesting as reduced normal sleep, increased number of awakenings and abnormal sleep.^1^,^2^ Surgical treatment is the most effective and direct way to treat OSAHS. However, surgical treatment is highly traumatic and restricted by factors such as parents’ fear.^3^ The research of Stewart^4^ et al has shown that sublingual immunotherapy can effectively control nasal allergy symptoms, sleep snoring symptoms and adenoid size in children. Montelukast can be used in combination with several medications that are commonly used for asthma prophylaxis and long-term treatment, as well as for seasonal allergic rhinitis. It was shown in the study of Chen H et al.^5^ that budesonide combined with Montelukast sodium has an accurate clinical efficacy in the treatment of allergic rhinitis, offering the advantages of effective relief of clinical symptoms, reduction of inflammatory response and high safety. Therefore, in this study, Montelukast combined with sublingual immunotherapy was used to treat children with OSAHS complicated with allergic rhinitis, and the efficacy was observed, providing a reference for clinical treatment of OSAHS complicated with allergic rhinitis.

## METHODS

This is a prospective study. A total of 102 children with OSAHS complicated with allergic rhinitis admitted to Hunan Children’s Hospital from April 2020 to April 2021 were selected and randomly divided into two groups according to the random number table method: sublingual group and two-drug combination group, with 51 cases in each group. Specifically, the random grouping method was as follows: (1) to draw up the serial numbers of 102 subjects in advance; (2) to generate random numbers (using random number table method); and (3) to specify that the subjects whose random numbers are odd are divided into sublingual administration group. even numbers were divided into two drugs combination group.

### Ethical Approval

The study was approved by the Institutional Ethics Committee of Hunan Children’s Hospital (No.: 2020091405; date: September 14,2020), and written informed consent was obtained from all participants.

### Inclusion criteria:

- Both groups of children were in line with the diagnostic criteria of “Diagnosis and Management of Allergic Rhinitis in Children”^6^ and “Diagnosis and Treatment of OSAHS in children”^7^,
- Both groups of children were in line with the indications and contraindications stipulated in “Expert Consensus on Allergen Specific Immunotherapy for Allergic Rhinitis (2011)”.^8^
- All Children and their families in this study were informed and signed the informed consent.

### Exclusion criteria:

- Children with severe heart, lung, kidney and liver dysfunction;
- Children without mental disorders or communication disorders;
- Children with incomplete case data;
- Children with immunodeficiency;
- Children with severe systemic diseases;
- All children allergic to the study drug.

### Drug source

Dust mite drops (Zhejiang Wolwo Bio-Pharmaceutical Co., Ltd.; State Drug Approval No.: S20060012), specification: 2ml; Montelukast (Sichuan Otsuka Pharmaceutical Co., Ltd., State Drug Approval No.: H20064370), specification: 10mg*5s.

### Treatment methods

Children in the sublingual treatment group were treated with standardized dust mite drops Nos. 1-4 for SLIT. At the 1st, 2nd, and 3rd weeks, droplet No. one at 1 μg/mL, Droplet No. two at 10 μg/mL, droplet No. three at 100 μg/mL were used in order of 1, 2, 3, 4, 5, 6, 7, 8, 9, 10 drops for one to seven days, and then droplet No. four at 333 μg/mL was used in three drops each time. All the children placed the drops under the tongue after brushing their teeth in the morning, swallowed them after four minutes, and fasted within 15 minutes of swallowing. They took the drops at a fixed time every day at 7-8 o’ clock in the morning for two years; In contrast, children in the two-drug combination group were treated with Montelukast at a 5mg oral dose for one month in addition to the sublingual group. Before the beginning of treatment and after the end of the course of treatment, monitoring of AHI and Lsao_2_ indexes: Sleep apnea hypopnea index (AHI) and hypoxic saturation (Lsao_2_) were monitored by polysomnography (PSG).

### Detection of IL-4 and IL-17 levels

Before the beginning of treatment and after the end of the course of treatment, the early morning venous blood of the two groups were taken, stored in a disposable anticoagulant tube, centrifuged with a 3000 r/min centrifuge, and after 10 minutes, the supernatant was separated and stored at -80°C. The levels of IL-4 and IL-17 were detected by chemiluminescence immunoassay.

### OSA-18 score, TNSS score

Before the beginning of treatment and after the end of the course of treatment, the OSA-18 Snoring Symptom Scale for children^9^ was used to score snoring symptoms, with reliability=8.59 and validity=9.11. The higher the score, the more severe the symptoms. The Allergic Rhinitis Symptom Scale (TNSS) was developed by referring to the “Diagnosis and Treatment Principles and Recommended Protocols for Allergic Rhinitis”, with reliability=8.88 and validity=9.13. The higher the score, the more severe the symptoms.

### Comparison of efficacy

Before the beginning of treatment and after the end of the course of treatment, the efficacy was divided according to the 2007 Draft Guidelines for the Diagnosis and Treatment of Children OSAHS (Urumqi). *Markedly effective:* Clinical symptoms disappeared basically, and AHI less than five times/h. *Effective:* clinical symptoms were significantly alleviated, and AHI decreased ≥25%. *Invalid:* No improvement in clinical symptoms, exacerbation of the disease, AHI reduction <25%. Total effective rate = (markedly effect + effective)/total number of cases×100%. *Comparison of the occurrence of adverse reactions:* The adverse reactions such as urticaria, rash, hallucinations, and excitement were counted in the two groups and compared.

### Statistical processing

All data in this study were analyzed and processed using SPSS 20.0 statistical software. Measurement data were described by mean ± standard deviation (*χ̅*±*S*), independent samples t-test was used for comparison between groups, repeated measures data were used for comparison before and after treatment, and repeated measures analysis of variance was performed; The count data were expressed by frequency and percentage, and the comparison between groups was by *x*^2^ test. P<0.05 was considered statistically significant.

## RESULTS

In the sublingual group, there were 26 males and 25 females, aged 3-16 years, with an average age of (9.03±6.98), a disease course of 2-9 months, and an average disease course of (5.22±3.71); In the two-drug combination group, there were 27 males and 24 females, aged 2-15 years, with an average age of (8.08±6.91), a disease course of 2-8 months, and an average disease course of (4.75±3.21); No statistically significant difference was observed between the two groups in gender, average age, average course of disease and other data (P>0.05), indicating comparability.

As shown in Table-I, no statistically significant difference was observed in the comparison of AHI and Lsao_2_ between the two groups before treatment (P>0.05). After treatment, the AHI index of the two-drug combination group was significantly decreased, and the Lsao_2_ index was significantly increased compared with the sublingual group, with statistically significant differences (P<0.05). Compared with the sublingual group, the AHI index of the two-drug combination group was significantly decreased, and the Lsao_2_ index was significantly increased, with a statistically significant difference (P<0.05).

**Table-I:** Comparison of AHI and Lsao_2_ indexes between the two groups before and after treatment (*χ̅*±*S*).

Group	Number of cases	AHI value		Lsao_2_ value	

		Before treatment	After treatment	Before treatment	After treatment
Sublingual group	51	25.15±4.15	11.45±2.15	75.95±9.89	81.15±6.11
Two-drug Combination group	51	25.46±4.22	7.15±1.23	76.89±10.45	87.89±5.45
*t*		0.374	12.400	0.467	5.879
*P*		0.709	0.001	0.642	0.001

No statistically significant difference was observed in the levels of IL-4 and IL-17 between the two groups before treatment (P>0.05). Tale-II After treatment, the levels of IL-4 and IL-17 in the two groups were significantly decreased, with a statistically significant difference (P<0.05). After treatment, compared with the sublingual group, the levels of IL-4 and IL-17 in the two-drug combination group were significantly decreased, with a statistical difference (P<0.05).

No statistically significant difference was observed in the comparison of OSA-18 score and TNSS score between the two groups before treatment (P>0.05). Table-III Compared with before treatment, the OSA-18 score and TNSS score of the two groups of children after treatment were significantly decreased, with a statistically significant difference (P<0.05). After treatment, the OSA-18 score and TNSS score of the children in the two-drug combination group were significantly lower than those in the sublingual group, with a statistically significant difference (P<0.05). Compared with the sublingual group, the efficacy of the two-drug combination group was significantly increased, with a statistically significant difference (P<0.05).Table-IV The incidence of adverse reactions in the two-drug combination group was significantly lower than that in the sublingual group, with a statistically significant difference (P<0.05). Table-V

**Table-II:** Comparison of IL-4 and IL-17 levels in the two groups before and after treatment (*χ̅*±*S*), ng/L.

Group	Number of cases	IL-4		IL-17	

		Before treatment	After treatment	Before treatment	After treatment
Sublingual group	51	205.45±12.45	118.45±15.45	7.89±1.25	3.56±0.33
Two-drug Combination group	51	206.45±13.25	106.15±16.23	7.99±2.15	1.15±0.15
*t*		0.393	3.920	0.287	47.480
*P*		0.695	0.001	0.775	0.001

**Table-III:** Comparison of OSA-18 score and TNSS score between the two groups before and after treatment (*χ̅*±*S*), points.

Group	Number of cases	OSA-18 score		TNSS score	

		Before treatment	After treatment	Before treatment	After treatment
Sublingual group	51	82.15±3.56	62.15±1.56	5.75±0.56	4.11±0.12
Two-drug Combination group	51	83.15±4.11	42.15±1.33	5.89±0.33	2.15±0.13
*t*		1.313	69.670	1.538	79.120
*P*		0.192	0.001	0.127	0.001

**Table-IV:** Comparison of efficacy between the two groups n (%).

Group	Number of cases	Markedly effective	Effective	Invalid	Total efficiency
Sublingual group	51	21 (41.18)	15 (29.41)	15 (29.41)	36 (70.59)
Two-drug combination group	51	29 (56.86)	18 (35.29)	4 (7.84)	47 (92.16)
*x^2^*					10.150
*P*					0.001

**Table-V:** Comparison of adverse reactions in two groups n (%).

Group	Number of cases	Urticaria	Rash	Hallucination	Excited	Total adverse reaction rate
Sublingual group	51	1 (1.96)	0 (0.00)	0 (0.00)	0 (0.00)	1 (1.96)
Two-drug Combination group	51	1 (1.96)	0 (0.00)	0 (0.00)	1 (1.96)	2 (3.92)
*x^2^*						0.365
*P*						0.546

## DISCUSSION

OSAHS is a common respiratory disease in children, which can mainly induce adenoid hypertrophy. Despite the fact that surgical treatment is more thorough, surgical treatment has poor compliance considering that children are younger and require general anesthesia during surgery.^10^ Allergic rhinitis may aggravate OSAHS symptoms in children by stimulating adenoid hyperplasia of the nasopharynx, and may also stimulate the occurrence and development of OSAHS in children via the immune response of the nose and throat.^11^ It was shown in the study^12^ that the SLIT boasts various benefits, such as effective control of nasal allergy symptoms in children with OSAHS complicated with AR, improvement of OSAHS symptoms and adenoid hypertrophy, and amelioration of prognosis. Our research has shown that Montelukast combined with sublingual immunotherapy boasts various advantages, such as effectively controlling nasal allergy symptoms in children with OSAHS complicated with allergic rhinitis, improving OSAHS symptoms and adenoid hypertrophy, and ameliorating the prognosis of children.

Sublingual immunotherapy is a treatment for allergic rhinitis, which continuously induces the natural immune regulation process through long-term intake of a small dose of allergen, and ultimately leads to clinical tolerance of patients.^13^ Relevant clinical studies^14^ have shown that sublingual immunotherapy has long-term efficacy, can significantly reduce the clinical symptoms of patients, and can effectively avoid the occurrence of new allergens. Sublingual immunotherapy has a long-term effect, and the effect is more pronounced as the course of treatment increases. However, it also has certain limitations, such as poor efficacy for pollen allergy and combined fungal allergens.^15^ Montelukast is a leukotriene receptor antagonist, which inhibits the inflammatory response caused by leukotriene by blocking the binding of leukotriene and its receptor, and achieves anti-inflammatory and anti-asthma effects.^16^

In this study, Montelukast combined with SLIT can significantly decrease the AHI index and increase the Lsao2 index in children with OSAHS complicated with allergic rhinitis. Indicating that the two-drug combination can effectively improve the clinical symptoms of children with significant effects, which is worthy of clinical application. According to the research results of Liu W et al.^17^, sublglossal immunotherapy have good efficacy and safety in the treatment of allergic rhinitis in children, which is worthy of clinical application and promotion. It was shown in the study by Lin JL et al.^18^ that Montelukast has a significant effect on patients with bronchial asthma complicated with OSAHS and can effectively reduce the levels of TNR-α, CRP, IL-6, AHI and FeNO, providing reference for clinical medication in elderly patients with bronchial asthma complicated with OSAHS. Ehsan Z et al.^19^ showed that intranasal hormone combined with Montelukast had a significant short-term effect in the treatment of OSAHS in children. It is consistent with the research results of this paper.

IL-4 can activate PTK and JAK kinases, enhance their antigen presentation ability, promote humoral immune response, and induce IgM to IgE transformation. In addition, IL-4 also up-regulates B-cell IgE transcription, enhances IgE mediated eosinophil degranulation, and exacerbates eosinophil mediated airway inflammation.^20^,^21^ As shown by Zhang Z et al.^22^, OSAHS in children has a close bearing on hypertension and serum inflammatory factor levels. Non-spoon blood pressure rate, body mass index and serum TNF-α, IL-4, IL-6 and IL-8 levels are positively correlated with the severity of OSAHS in children, while nocturnal blood pressure drop rate is negatively correlated with the severity of OSAHS in children. IL-17 mainly exists in lymphatic tissues and can promote the differentiation of neutrophils and accumulate in lung tissue, leading to airway inflammation and participating in the pathogenesis of OSAHS. In the study of Ma CY et al.^23^, the results showed that patients with moderate and severe OSAHS had abnormal left ventricular function and the activation of inflammatory response and oxidative stress response was related to the change of left ventricular function. It was found in this study that the levels of IL-4 and IL-17 in the two-drug combination group were significantly reduced, indicating that the combination of the two drugs could effectively reduce the levels of IL-4 and IL-17 in the children with a significant effect. It was also found in this study that the OSA-18 score and TNSS score of children in the two-drug combination group were significantly reduced, indicating that the two-drug combination could significantly reduce the symptom score of children with significant effect. According to the study by Sun W et al.^24^, Montelukast and budesonide has a significant therapeutic effect on patients with cough variant asthma and can effectively reduce the expression of inflammatory factors in patients, which is feasible. Kuhle S et al.^25^ showed that anti-inflammatory treatment can significantly improve PSG index, adenoid and quality of life in children with OSAHS, especially in children with mild OSAHS.

### Limitations of this study

It includes small sample size of this study is small, the clinical data is limited, and the persuasive conclusions are also limited. Trial with large sample size are needed to confirm our observations.

## CONCLUSION

Montelukast combined with sublingual immunotherapy offers various advantages, such as effectively controlling nasal allergy symptoms in children with OSAHS complicated with allergic rhinitis, improving OSAHS symptoms and adenoid hypertrophy, and ameliorating the prognosis of children.

### Authors’ Contributions:

**XM:** Designed this study and prepared this manuscript.

**WZ:** Collected and analyzed clinical data and significantly revised this manuscript.
